# Climatic effects on water quality in areas with acid sulfate soils with commensurable consequences on the reproduction of burbot (*Lota lota* L.)

**DOI:** 10.1007/s10653-020-00550-1

**Published:** 2020-03-17

**Authors:** Janne Toivonen, Richard Hudd, Miriam Nystrand, Peter Österholm

**Affiliations:** 1 Department of Geology and Mineralogy, Åbo Akademi University, Akademigatan 1, 20500 Åbo, Finland; 2 Vaasa, Finland

**Keywords:** Acid sulfate soils, Extreme variable water quality, Reproduction of fish, Bioindicator

## Abstract

Due to discharge from acid sulfate (a.s.) soils, watercourses and coastal areas in the Gulf of Bothnia are periodically heavily acidified with high concentrations of potentially toxic metals. Data on water quality from 2005 to 2014 in an embanked lake, an estuary of four rivers in western Finland, showed repeated events with acidic water (pH < 5.5) with high concentrations of Al. Size fractionation and species modeling of Al showed that a significant part of the Al occurred as highly toxic small-size fractions (dissolved < 1 kDa and colloidal 1 kDa—0.45 µm) as free ions and complexed to sulfate. The larval abundance of the burbot (*Lota lota* L.) was shown to be sensitive to acidity during the wintertime spawning migration and spawning. Bearing in mind the importance of estuaries of the northern Baltic Sea as spawning and nursery areas of fish, the reoccurring failure in the reproduction of fish may cause a more serious threat for the lake and adjacent coastal fish stocks than the spectacular, but less frequent, mass kills of adult fish. This demonstrates the close relationship between climate, hydrology, water geochemistry and the aquatic coastal ecosystem in areas affected by a.s. soils. As the current forecast of climate chance indicates warmer winters with more continuous runoff, the effects can become even more prominent. This study also shows that the annual larvae abundance of burbot may be used as a bioindicator and an instrument for the fisheries for obtaining more comprehensive knowledge of the ecological effects of acidic metal discharge from a.s. soils.

## Introduction

Acid sulfate (a.s.) soils are found in areas covering over 17 million ha (Andriesse and van Meensvoort 2006) and can cause serious ecological problems in the form of high acidity and high concentrations of potentially toxic metals in nearby water courses. Acid sulfate soils in Finland, and along the coastal zone of the Baltic Sea in general, originate from the sulfide-rich sediments that were deposited during the Littorina and Post-Littorina Sea (7500—0 BP) stages of the Baltic Sea. Due to post-glacial land uplift (currently up to 8 mm per year), these sediments occur in coastal areas, 0–100 m above sea level (commonly 0–60 m, Erviö 1975; Palko 1994). When drained, the sulfides in the sediments oxidize, aided by microbial activities, to sulfuric acid. Consequently, the sediments develop into an a.s. soil (pH < 4). In this acidic environment, many potentially toxic metals (e.g., Al, Cd, Co, Ni and Zn) dissolve from the silicate minerals, mainly phyllosilicates (Åström and Björklund 1997; Deng et al. 1998). Therefore, acidic water with high concentrations of sulfate and metals is flushed to nearby watercourses during rainfall and snowmelt (Åström and Björklund 1995; Macdonald et al. 2007; Österholm and Åström 2002). Discharge of acidity and metals from a.s. soils is the main reason why most of the rivers in mid-western Finland have a poor chemical status, which has caused widespread ecological disturbance not only in rivers, but also in coastal areas (e.g., Hildén et al. 1982; Hudd and Leskelä 1998; Wallin et al. 2015). Visible mass kills of fish related to acidic metal discharge has been recorded already in the nineteenth century (Sutela et al. 2012; Suupohja et al. 1973) when many shallow lakes were drained for land reclamation. Problems with permanently poor water quality and declining fish stocks started in the 1960s and 1970s because of intensive drainage projects and the replacing of existing open drains with effective subsurface drainage systems (Åström et al. 2005; Hildén and Rapport 1993; Saarinen et al. 2010; Suupohja et al. 1973; Toivonen et al. 2013, 2019).

Mass kills of adult fish is a spectacular result of acidic metal discharge in areas with a.s. soils. However, there are more significant and frequently occurring consequences; estuaries namely offer important reproduction sites for fish in boreal environments due to favorable temperatures and high amount of available shelter and food, but are at the same time vulnerable to the acidic metal discharge carried by the rivers (Lehtonen and Hudd 1990). The spawning and early life stages of fish are more sensitive to acidic events, causing frequent failure in offspring production with no immediate obvious effects. Instead, the effects appear as declining and abnormally fluctuating fish stocks and difficulties for fish stocks to recover between visible mass kills of fish (Böhling et al. 1991; Hudd et al. 1986; Hudd and Kjellman 2002; Kjellman et al. 1994; Sammut et al. 1995; Sayer et al. 1993). Since fish of several species from large areas along the coast use estuaries for reproduction, the effects on fish populations are widespread (Sutela et al. 2012). Consequently, together with the fact that acidic events are often short and intensive (Österholm and Åström 2008; Toivonen et al. 2013, 2019), sufficient knowledge about the full extent of such events in rivers, and especially in estuaries and coastal areas, is not gained with just occasional water sampling.

The adult coastal burbot (*Lota lota* L.) show a cold stenothermal biology (Tiitu and Vornanen 2002), meaning that they are only found in spawning areas in rivers and estuaries during the winter and dwell in the sea during the rest of the year where temperatures are more stable and cooler (Fisher and Eckmann 1997; Hudd et al. 1983, Hudd et al. 2007). The young of the year stay in shallow near-shore waters throughout the first summer (Eloranta 1985; Fisher and Eckmann 1997). In the study area, the burbot migrate from the sea to the lake in late December and spawns in early or mid-February (pers. comm. with local fishermen), supported by taggings (Hudd and Lehtonen 1987). Hatching takes place close to the ice breakup in April. After hatching and distribution pelagically, the larvae actively migrate to the shallowest shores with typical habitats of floating dead reed (*Phragmites australis*). Since the spawning and the embryonic and early larval development of fish is often more sensitive to the acidic metal load than the adult, the place and timing of the critical stages of the reproduction causes the recruitment of the burbot to be highly exposed to the acidic metal load carried by the rivers and streams.

The aims of this study are to examine

- how meteorologically driven hydrological variations in a catchment with a.s. soils affect the water quality in the coastal recipient lake.
- how these factors explain the reproduction success of coastal fish with focus on the commercially interesting fish burbot (*Lota lota* L.), and
- the possibility of using the abundance of burbot larvae as a bioindicator on the annual varying effects of discharge from a.s. soils.

## Research area

**Fig. 1:**
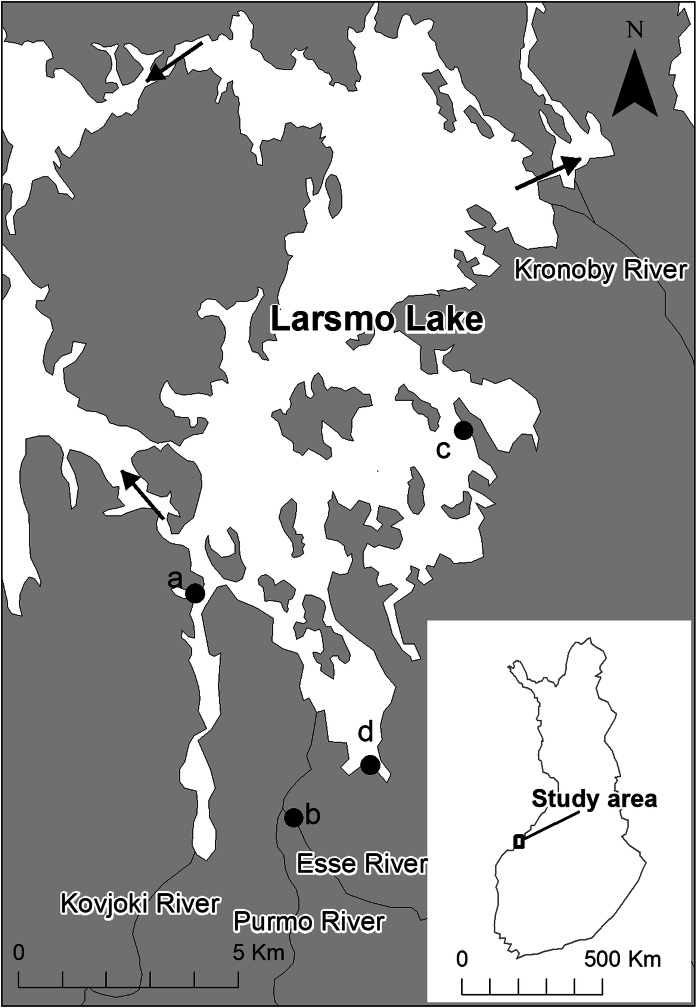
Location of the Larsmo Lake, water-quality monitoring sites (a: Larsmo Lake, b: Esse River), outlets (arrows) and the sites for burbot larvae samplings (c and d)

Larsmo Lake (73 km^2^, Fig. 1) is an estuary of four rivers (names of the rivers in Fig. 1) and was embanked from the Baltic Sea in the 1960s for industrial needs (freshwater reservoir). Mainly because of recreational needs, any great fluctuations in the water level have been prevented since 1998 by regulating the outlets from the lake, and the water level is set at 60 cm above normal seawater level. The lake acts as an important spawning area for the burbot, and many other fish species reproducing in estuaries, and constructed fish ways allows fish migration to and from the sea. The lake is connected to the nearby Öja Lake (also an embanked freshwater reservoir) via a canal in the northeast. Even though both lakes are often considered as one, this study focused only on Larsmo Lake. The drainage areas of the four rivers discharging into the lake consist of 14–19% arable land and of 20–34% peat land, and the land use is dominated by forestry and agriculture. Since the late 1960s, the rivers and the lake have suffered many acidic events with mass kills of fish and declining fish stocks (Palko and Alasaarela 1988; Sutela et al. 2012). An increase in the exploitation of a.s. soils, and perhaps to some extent, a minor change in climate, together with the inhibited dilution and neutralization by alkaline brackish water by the embankments are the major reasons for the water-quality problems in the lake (Snickars and Wistbacka 2000; Toivonen et al. 2013, 2019). The a.s. soils in western Finland are present mainly in the stream and river valleys and consist mainly of clay/silt sediments (Åström and Björklund 1995). A map of the whole drainage area of the lake is published in Toivonen et al. 2019, and the location of a.s. soils in the study area can be found on the acid sulfate soil map service provided by the Geological Survey of Finland (http://en.gtk.fi/).

In contrast to most coastal areas in the world, tides at the Gulf of Bothnia are negligible, and variance at seawater level is relatively small. Therefore, there is no intrusion of brackish water into watercourses. Consequently, changes in electric conductivity (EC) and sulfate concentration in areas with a.s. soils are controlled by leaching from a.s. soils (Österholm and Åström 2008; Roos and Åström 2005; Toivonen et al. 2013).

Even though many toxic elements are flushed to watercourses from a.s. soils, Al is considered to be the most hazardous metal to aquatic life in acidic conditions due to the great abundance in soil minerals (third most abundant crustal element), acid solubility and great biological effects (Fältmarsch et al. 2008; Gensemer and Playle 1999). Since the main data on Al used in this study represent total concentrations, Al may partly be caused by erosion and occur in the particulate fraction (> 0.45 µm, Nystrand et al. 2012). However, studies on a.s. soils in western Finland (Åström and Björklund 1995; Nystrand and Österholm 2013; Roos and Åström 2005; Toivonen and Österholm 2011) together with the flat topography and the lack of the characteristic turbid water typical of erosion, strongly suggest that erosion is not an important source of Al.

## Materials and methods

### Statistical methods

Medians and percentiles of available parameters were used in the study in order to reduce the effects of potential errors and outliers. Spearman rank correlation (*p *= 0.05) was used for correlations due to the high probability of skewed distributions (Helsel and Hirsch 2002; Reimann and Filzmoser 1999).

### Water quality

Data on water quality in the lake were provided by UPM Kymmene paper mill in Jakobstad (water intake lies at point a, Fig. 1) and included measurements of pH and electric conductivity (EC) every working day. Aluminum (photometric analysis of acid soluble Al) and sulfate (high-performance liquid chromatography (HPLC)) concentrations were analyzed once or twice per week. The data used in this study also included the main river, the Esse River (accounts for about 48% of the water discharge to the lake), and was provided by the Jakobstad water plant (water intake lies at point b, Fig. 1). The data on the river include pH measured every day.

Daily data on runoff from an unregulated stream were obtained from the national monitoring database (www.syke.fi/avointieto). The site is located approximately 28 km northeast from the study area.

Water sampling was performed by the authors in the lake (close to site a, Fig. 1) and the Esse River (site b, Fig. 1) during autumn flood conditions 2013. The particulate (> 0.45 µm), colloidal (1 KDa—0.45 µm) and dissolved (< 1 KDa) fractions of Al were analyzed according to Nystrand et al. (2012). See description of the method in Nystrand et al. (2012).

Factors and elements known to affect Al species are temperature, pH, fluoride (F), organic matter (OM), sulfate, Cl, Na, Mg, K and phosphate (Nystrand and Österholm, 2013). The species of Al in the study area was assessed by geochemical modeling using Visual MINTEQ (vers. 3.0; Gustafsson 2010). The data used in the modeling of Al species were based on different acidic circumstances (pH 4.5–6.0) and corresponding sulfate (29–75 mg/l, respectively) and Al concentrations (0.9–3.7 mg/l, respectively) found in the data from the paper industry (2005–2014). No data on F were available. Fluoride was therefore analyzed with ion chromatography (Dionex DX-120) from the lake sample (close to site a, Fig. 1). The concentrations of OM (Shimadzu Organic Carbon 5050 analyzer), Cl (HPLC), Na, Mg, K and phosphate (inductively coupled plasma-optical emission spectrometry and inductively coupled plasma mass spectrometry) used in the modeling were based on analyses from lake samples (Toivonen, unpublished data) which were considered to represent the lake water during the different acidic circumstances. See description of the method in Nystrand and Österholm (2013).

### Occurrence of burbot larvae

Potential habitats and larval areas for the early life stages of burbot in the study area were mapped and modeled by Hudd et al. (2007). The only variable explaining the occurrence of burbot larvae within the lake was the availability of optimal habitat (shallow water with floating 1–2-year-old common reed, *Phragmites australis*). The occurrence of burbot larvae was measured with a simple method demonstrated by Johnsson, (Researcher at Umeå University, pers. comm.) and developed by Hudd et al. (1983). For this study, the sampling of burbot larvae was performed during 10 years (2005–2015, with 2010 missing) at site c, and 9 years (2006–2015, with 2010 missing) at site d (Fig. 1). The samples were taken with a 1 l plastic scoop in optimal habitats. Because the period when the larvae are easily caught with the method is short due to larval growth and to the successive change to bottom life, sampling results later than 15 May were therefore excluded. 29–186 samples per site were taken during the first half of May each year, and the probability of capturing any larvae per sample was used as an index of abundance (hit rate).

To detect periods crucial for the abundance of burbot larvae, the time preceding the sampling was divided into 10-day intervals, as well as months, for which water-quality statistics (10th and 90th percentiles and medians for pH, Al and sulfate, and number of days with pH below different threshold values) based on water-quality data from the lake were calculated. These water-quality statistics were correlated against the hit rate.

## Results

### Water quality

**Fig. 2:**
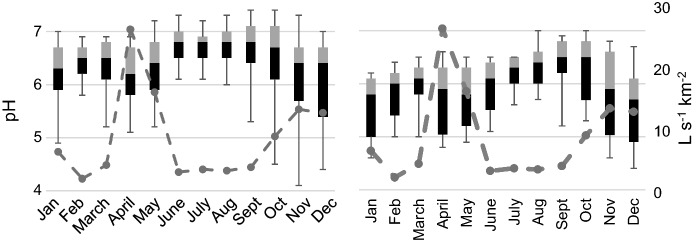
Minimum (lower whisker), 10th percentile (black), median, 90th percentile (gray) and maximum (upper whisker) pH measured in Esse River (left) and Larsmo Lake (right) 2005–2014. The dotted line indicates the average runoff (L s^−1^ km^−2)^ for each month

**Fig. 3:**
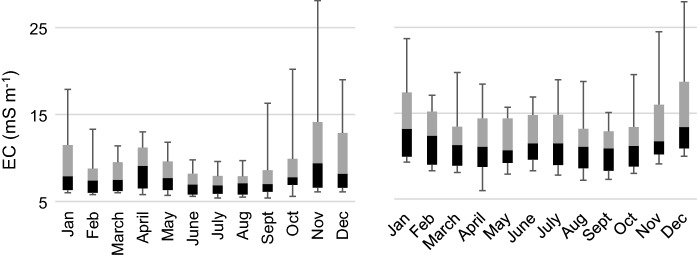
Minimum (lower whisker), 10th percentile (black), median, 90th percentile (gray) and maximum (upper whisker) EC measured in Esse River (left) and Larsmo Lake (right) 2005–2014

**Fig. 4:**
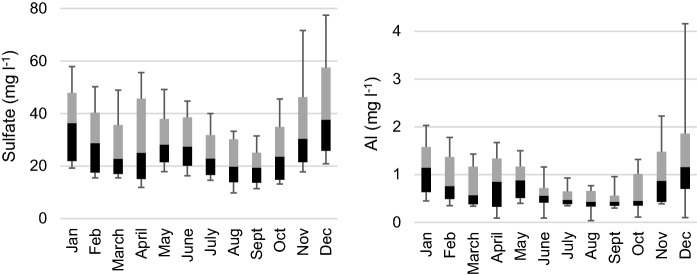
Minimum (lower whisker), 10th percentile (black), median, 90th percentile (gray) and maximum (upper whisker) sulfate (left) and Al concentrations (right) measured in the Larsmo Lake 2005–2014

During the 10-year study period (2005–2014), acidic water (pH < 5.5) in the Esse River was observed during 4 years, while very acidic water (pH < 5.0) was observed during 3 years. Acidic water in the recipient Larsmo Lake was observed during nine out of ten years, and very acidic during three out of ten years. The median pH was during the different months over the whole year 6.2–6.8, and the 10th percentile pH was 5.4–6.5 in the Esse River, and somewhat lower in the Larsmo Lake: 5.7–6.5 and 4.9–6.2, respectively (Fig. 2). Median pH in the river was the lowest in April (median pH 6.2), while the lowest minimum pH was found in December (10th percentile pH 5.4), followed by November, April, January and May. The overall most acidic month in the lake was December (10th percentile and median pH 4.9 and 5.9, resp.), followed by January, April, May and November. Typical for areas with a.s. soils, EC and concentrations of sulfate and Al were overall high, peaking during months with the lowest pH (Figs. 3, 4).

### The geochemical modeling and size distribution of Al

**Fig. 5:**
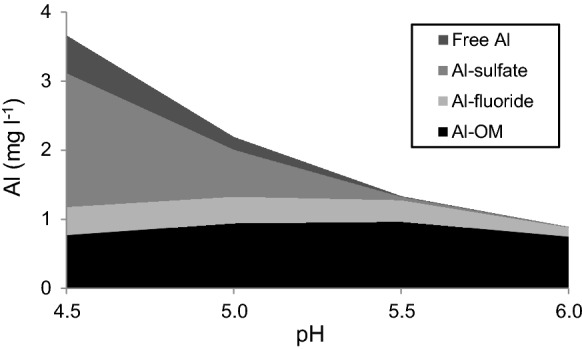
The distribution of Al species based on different acidic conditions found 2005–2014 in available data occurring as Al^3+^ (Free Al), sulfate (Al-sulfate), fluoride (Al-fluoride) and organic matter (Al-OM)

**Fig. 6:**
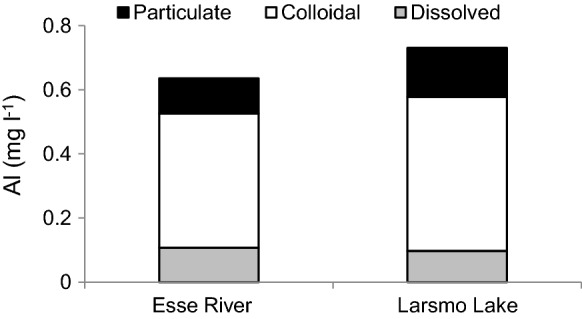
The size distribution of Al in water samples from the Esse River and Larsmo Lake during autumn flood conditions 2013

The total Al concentrations were the highest at low pH and showed a clear decrease with increasing pH (Fig. 5). The results from the modeling of the distribution of the Al species showed that concentrations of Al complexed to sulfate and free Al ions dominated at pH 4.5, but decreased with increasing pH and were absent at pH > 5.5. The absolute amounts of Al complexed to organic matter (OM) and F were relatively constant over the range (pH 4.5–6.0). The former complex became dominant at pH > 5.0. The modeling of the Al species was performed on total concentrations and the particulate fraction during all pH-levels was unknown and unaccounted for. However, the importance of the particulate fraction is expected to be low during acidic circumstances (Fig. 6 and Nystrand et al. 2012). The water samples analyzed for the size distribution of Al had a pH of 5.5 (Esse River) and 5.6 (Larsmo Lake). Electric conductivity was 10 and 12 mS/m, and sulfate concentrations were 24 and 30 mg/l, respectively. The colloidal fraction was the dominating species, but particulate and dissolved fractions were also present (Fig. 6).

### Impact of acidity on the abundance of burbot larvae

**Fig. 7:**
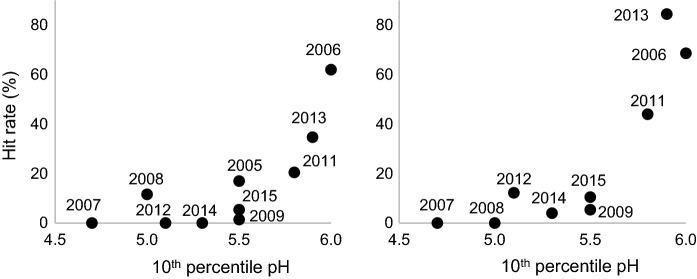
10th percentile pH in Larsmo Lake during January versus hit rate at site c (left) and site d (right)

The period that appeared to be important in explaining the hit rate at both site c and d seemed to take place during the winter. 10th percentile and median pH showed significant correlations for January (*r*_s_ up to 0.87) and February (*r*_s_ up to 0.78, Fig. 7 and Table 1). The hit rate was high in 2006, 2011 and 2013 at both sites (21–62% for site c and 44–85% at site d), corresponding to higher pH, while low abundance (hit rate < 20%) or total absence of burbot larvae was observed during the remaining years. The number of days with pH < 5.9 (data not shown) and pH < 6.0 based on the ten-day periods, as well as 10th percentile and median pH, spanning from late December to mid-February or early-March at both sites explained the abundance well (r_s_ up to 0.93, Table 2). Aluminum- or sulfate concentrations did not seem to be able to explain the hit rate. Elevated runoff (average runoff > 5 L s^−1^ km^−2^) in January caused lowered abundance of burbot larvae at site d (*r*_s_ = − 0.89, data not shown), but not at site c.

**Table 1:** 10th percentile pH in Larsmo Lake during January versus hit rate at site c (left) and site d (right)

	Site c		Site d	
	10th perc. pH	Med pH	10th perc. pH	Med pH
Dec	0.61	0.52	**0.71**	0.49
Jan	**0.83**	**0.80**	**0.87**	**0.87**
Feb	**0.78**	**0.69**	**0.75**	**0.73**
March	0.58	0.57	**0.77**	**0.78**
April	0.02	0.25	0.08	0.29

Values in bold are significant correlations

**Table 2:** Water-quality statistics (10th percentile, median pH and number of days with pH < 6.0) versus hit rate during 10-day periods at site c (left) and site d (right)

Date	Site c			Site d		
	10th perc pH	Median pH	Number of days with pH < 6.0	10th perc. pH	Median pH	Number of days with pH < 6.0
Dec-01	0.17	0.19	− 0.07	0.35	0.31	− 0.13
Dec-02	0.17	0.23	− 0.06	0.35	0.31	− 0.13
Dec-03	0.20	0.30	− 0.29	0.35	0.32	− 0.10
Dec-04	0.41	0.31	− 0.32	0.47	0.44	− 0.10
Dec-05	0.42	0.31	− 0.31	0.48	0.44	− 0.10
Dec-06	0.42	0.41	− 0.42	0.48	0.48	− 0.21
Dec-07	0.42	0.37	− 0.42	0.48	0.45	− 0.21
Dec-08	0.42	0.37	− 0.42	0.48	0.45	− 0.21
Dec-09	0.42	0.37	− 0.42	0.48	0.45	− 0.21
Dec-10	0.33	0.37	− 0.42	0.41	0.45	− 0.21
Dec-11	0.24	0.37	− 0.36	0.34	0.45	− 0.16
Dec-12	0.24	0.40	− 0.34	0.34	0.46	− 0.16
Dec-13	0.26	0.35	− 0.34	0.38	0.42	− 0.16
Dec-14	0.45	0.40	− 0.34	0.55	0.47	− 0.16
Dec-15	0.48	0.40	− 0.34	0.56	0.47	− 0.16
Dec-16	0.56	0.40	− 0.34	0.63	0.47	− 0.16
Dec-17	0.56	0.50	− 0.34	0.63	0.56	− 0.16
Dec-18	0.56	0.52	− 0.34	0.68	0.60	− 0.16
Dec-19	0.58	0.56	− 0.34	0.68	0.63	− 0.16
Dec-20	0.58	0.57	− 0.34	0.68	0.66	− 0.16
Dec-21	**0.79**	**0.83**	− 0.36	0.66	**0.71**	− 0.16
Dec-22	**0.79**	**0.86**	− 0.49	0.66	**0.72**	− 0.25
Dec-23	**0.83**	**0.88**	− 0.49	**0.69**	**0.76**	− 0.25
Dec-24	**0.83**	**0.90**	**−** **0.76**	**0.75**	**0.74**	**−** **0.71**
Dec-25	**0.79**	**0.93**	**−** **0.73**	**0.82**	**0.77**	**−** **0.71**
Dec-26	**0.79**	**0.93**	**−** **0.73**	**0.81**	**0.77**	**−** **0.71**
Dec-27	**0.79**	**0.89**	**−** **0.73**	**0.81**	**0.71**	**−** **0.71**
Dec-28	**0.79**	**0.79**	**−** **0.73**	**0.81**	**0.81**	**−** **0.71**
Dec-29	**0.79**	**0.81**	**−** **0.73**	**0.82**	**0.84**	**−** **0.71**
Dec-30	**0.83**	**0.81**	**−** **0.75**	**0.84**	**0.84**	**−** **0.71**
Dec-31	**0.82**	**0.78**	**−** **0.73**	**0.84**	**0.84**	**−** **0.71**
Jan-01	**0.80**	**0.80**	**−** **0.73**	**0.84**	**0.85**	**−** **0.71**
Jan-02	**0.80**	**0.80**	**−** **0.73**	**0.84**	**0.86**	**−** **0.71**
Jan-03	**0.82**	**0.78**	**−** **0.73**	**0.86**	**0.84**	**−** **0.71**
Jan-04	**0.82**	**0.80**	**−** **0.73**	**0.86**	**0.84**	**−** **0.71**
Jan-05	**0.80**	**0.80**	**−** **0.71**	**0.86**	**0.84**	**−** **0.71**
Jan-06	**0.82**	**0.82**	**−** **0.71**	**0.86**	**0.86**	**−** **0.71**
Jan-07	**0.82**	**0.82**	**−** **0.71**	**0.86**	**0.86**	**−** **0.71**
Jan-08	**0.81**	**0.81**	**−** **0.83**	**0.87**	**0.87**	**−** **0.79**
Jan-09	**0.81**	**0.80**	**−** **0.65**	**0.87**	**0.88**	− 0.53
Jan-10	**0.81**	**0.80**	**−** **0.65**	**0.87**	**0.87**	− 0.53
Jan-11	**0.81**	**0.80**	**−** **0.65**	**0.87**	**0.92**	− 0.53
Jan-12	**0.81**	**0.80**	**−** **0.83**	**0.87**	**0.92**	**−** **0.79**
Jan-13	**0.80**	**0.79**	**−** **0.83**	**0.88**	**0.90**	**−** **0.79**
Jan-14	**0.80**	**0.79**	**−** **0.83**	**0.93**	**0.90**	**−** **0.79**
Jan-15	**0.84**	**0.80**	**−** **0.84**	**0.87**	**0.87**	**−** **0.79**
Jan-16	**0.80**	**0.80**	**−** **0.84**	**0.87**	**0.87**	**−** **0.79**
Jan-17	**0.78**	**0.80**	**−** **0.85**	**0.87**	**0.87**	**−** **0.79**
Jan-18	**0.78**	**0.82**	**−** **0.86**	**0.87**	**0.87**	**−** **0.79**
Jan-19	**0.78**	**0.79**	**−** **0.88**	**0.87**	**0.87**	**−** **0.79**
Jan-20	**0.77**	**0.79**	**−** **0.88**	**0.85**	**0.89**	**−** **0.79**
Jan-21	**0.79**	**0.78**	**−** **0.88**	**0.85**	**0.87**	**−** **0.79**
Jan-22	**0.79**	**0.78**	**−** **0.81**	**0.85**	**0.87**	**−** **0.79**
Jan-23	**0.76**	**0.79**	**−** **0.81**	**0.85**	**0.87**	**−** **0.79**
Jan-24	**0.76**	**0.79**	**−** **0.74**	**0.85**	**0.87**	**−** **0.81**
Jan-25	**0.76**	**0.78**	**−** **0.75**	**0.85**	**0.87**	**−** **0.83**
Jan-26	**0.80**	**0.78**	**−** **0.75**	**0.87**	**0.87**	**−** **0.83**
Jan-27	**0.80**	**0.78**	**−** **0.75**	**0.88**	**0.87**	**−** **0.84**
Jan-28	**0.80**	**0.78**	**−** **0.75**	**0.88**	**0.87**	**−** **0.84**
Jan-29	**0.77**	**0.74**	**−** **0.75**	**0.86**	**0.83**	**−** **0.84**
Jan-30	**0.68**	**0.78**	**−** **0.73**	**0.86**	**0.75**	**−** **0.83**
Jan-31	**0.73**	**0.78**	**−** **0.70**	**0.85**	**0.75**	**−** **0.80**
Feb-01	**0.67**	**0.78**	− 0.63	**0.78**	**0.75**	**−** **0.73**
Feb-02	**0.78**	**0.83**	**−** **0.79**	**0.77**	**0.72**	**−** **0.82**
Feb-03	**0.78**	**0.76**	**−** **0.77**	**0.77**	0.66	**−** **0.82**
Feb-04	**0.78**	**0.68**	**−** **0.77**	**0.77**	0.67	**−** **0.82**
Feb-05	**0.78**	**0.68**	**−** **0.74**	**0.77**	0.68	**−** **0.79**
Feb-06	**0.80**	**0.78**	**−** **0.76**	**0.71**	**0.77**	**−** **0.82**
Feb-07	**0.68**	**0.76**	**−** **0.67**	0.68	**0.75**	**−** **0.74**
Feb-08	0.62	**0.76**	**−** **0.69**	0.68	**0.75**	**−** **0.76**
Feb-09	**0.67**	**0.76**	**−** **0.69**	**0.76**	**0.75**	**−** **0.76**
Feb-10	**0.73**	**0.79**	**−** **0.69**	**0.76**	**0.77**	**−** **0.76**
Feb-11	**0.80**	**0.75**	**−** **0.69**	**0.77**	**0.76**	**−** **0.76**
Feb-12	**0.72**	**0.75**	− 0.63	**0.77**	**0.78**	**−** **0.76**
Feb-13	**0.72**	**0.69**	− 0.63	**0.77**	0.68	**−** **0.76**
Feb-14	**0.70**	**0.69**	− 0.61	**0.76**	0.68	**−** **0.75**
Feb-15	**0.68**	**0.69**	− 0.59	**0.75**	0.68	**−** **0.74**
Feb-16	**0.68**	**0.69**	− 0.62	**0.75**	0.68	**−** **0.74**
Feb-17	**0.68**	**0.69**	− 0.62	**0.75**	0.68	**−** **0.74**
Feb-18	**0.70**	**0.67**	− 0.62	**0.75**	0.67	**−** **0.74**
Feb-19	**0.70**	**0.67**	− 0.62	**0.75**	0.67	**−** **0.74**
Feb-20	**0.71**	**0.67**	**−** **0.65**	**0.75**	0.67	**−** **0.74**
Feb-21	**0.68**	**0.67**	− 0.62	**0.75**	0.67	**−** **0.74**
Feb-22	**0.71**	0.62	− 0.48	**0.75**	**0.75**	− 0.62
Feb-23	**0.70**	0.57	− 0.48	**0.73**	**0.75**	− 0.62
Feb-24	**0.70**	0.57	− 0.62	**0.73**	**0.75**	**−** **0.74**
Feb-25	0.55	0.59	**−** **0.68**	0.67	**0.78**	**−** **0.74**
Feb-26	0.55	0.60	**−** **0.72**	0.67	**0.74**	**−** **0.69**
Feb-27	0.55	0.60	**−** **0.71**	0.67	**0.74**	− 0.65
Feb-28	0.55	0.57	**−** **0.71**	0.67	**0.70**	− 0.65
March-01	0.55	0.57	**−** **0.71**	**0.70**	**0.73**	− 0.65
March-02	0.62	0.59	**−** **0.76**	**0.75**	**0.73**	− 0.62
March-03	0.56	**0.68**	**−** **0.76**	**0.71**	0.62	− 0.62
March-04	0.56	**0.68**	**−** **0.76**	**0.71**	0.62	− 0.62
March-05	0.54	**0.68**	**−** **0.69**	**0.73**	0.62	− 0.49
March-06	0.31	**0.68**	**−** **0.68**	0.30	0.52	− 0.43
March-07	0.36	**0.68**	**−** **0.65**	0.23	0.52	− 0.36
March-08	0.36	**0.68**	**−** **0.65**	0.20	0.52	− 0.36
March-09	0.35	**0.68**	**−** **0.65**	0.30	0.52	− 0.36
March-10	0.34	**0.68**	**−** **0.68**	0.45	0.52	− 0.43
March-11	0.33	**0.65**	**−** **0.69**	0.49	0.55	− 0.49
March-12	0.32	0.58	**−** **0.67**	0.49	0.62	− 0.50
March-13	0.40	0.53	− 0.64	0.54	0.65	− 0.49
March-14	0.33	0.50	− 0.56	0.49	0.65	− 0.45
March-15	0.41	0.46	− 0.56	0.62	0.67	− 0.45
March-16	0.44	0.46	− 0.56	0.65	**0.74**	− 0.62
March-17	0.47	0.46	− 0.56	**0.81**	**0.77**	− 0.62
March-18	0.46	0.42	− 0.56	**0.81**	**0.74**	− 0.62
March-19	0.47	0.42	− 0.56	**0.79**	**0.73**	− 0.62
March-20	0.51	0.51	− 0.56	**0.79**	**0.77**	− 0.62
March-21	0.52	0.52	**−** **0.66**	**0.79**	**0.73**	**−** **0.73**
March-22	0.51	0.52	− 0.62	**0.74**	**0.73**	**−** **0.71**
March-23	0.47	0.52	− 0.62	**0.74**	**0.72**	**−** **0.71**
March-24	0.51	0.51	**−** **0.71**	**0.74**	**0.76**	**−** **0.75**
March-25	0.55	0.52	**−** **0.68**	**0.71**	**0.72**	**−** **0.74**
March-26	0.60	0.54	**−** **0.68**	0.64	0.68	**−** **0.74**
March-27	0.61	0.56	**−** **0.68**	0.64	0.69	**−** **0.74**
March-28	0.61	0.61	**−** **0.68**	0.64	0.61	**−** **0.74**
March-29	0.61	0.61	− 0.53	0.62	0.61	− 0.62
March-30	0.60	0.60	− 0.53	0.59	0.60	− 0.62
March-31	0.60	0.59	− 0.53	0.59	0.60	− 0.62
Apr-01	0.60	0.60	− 0.53	0.59	0.60	− 0.62
Apr-02	0.60	0.59	− 0.44	0.59	0.60	− 0.55
Apr-03	0.57	0.59	− 0.44	0.59	0.60	− 0.55
Apr-04	0.56	0.54	− 0.44	0.60	0.55	− 0.55
Apr-05	0.52	0.52	− 0.37	0.55	0.55	− 0.55
Apr-06	0.54	0.46	− 0.37	0.57	0.52	− 0.55
Apr-07	0.58	0.46	− 0.37	0.59	0.52	− 0.55
Apr-08	0.58	0.48	− 0.42	0.59	0.48	− 0.56
Apr-09	0.37	0.37	− 0.45	0.39	0.39	− 0.56
Apr-10	0.41	0.37	− 0.48	0.39	0.39	− 0.56
Apr-11	0.41	0.39	− 0.38	0.39	0.39	− 0.45
Apr-12	0.35	0.41	− 0.38	0.32	0.39	− 0.45
Apr-13	0.33	0.41	− 0.31	0.32	0.39	− 0.38
Apr-14	0.26	0.41	− 0.31	0.25	0.39	− 0.38
Apr-15	0.22	0.41	− 0.33	0.17	0.39	− 0.38
Apr-16	0.25	0.33	− 0.25	0.18	0.32	− 0.32
Apr-17	0.07	0.24	− 0.28	0.11	0.21	− 0.29
Apr-18	0.07	0.22	− 0.26	0.08	0.17	− 0.29
Apr-19	− 0.01	0.18	− 0.20	0.06	0.17	− 0.20
Apr-20	− 0.03	0.14	− 0.06	− 0.08	0.14	0.02
Apr-21	0.01	0.06	− 0.06	− 0.08	0.03	0.02
Apr-22	0.04	0.02	0.01	− 0.07	0.03	0.10
Apr-23	0.04	0.04	0.01	− 0.07	− 0.03	0.10
Apr-24	− 0.12	0.02	0.01	− 0.17	− 0.09	0.10
Apr-25	0.02	0.17	0.26	− 0.18	− 0.03	0.32
Apr-26	− 0.08	− 0.06	0.30	− 0.25	− 0.16	0.31
Apr-27	− 0.04	− 0.07	0.32	− 0.27	− 0.21	0.29
Apr-28	− 0.04	− 0.07	0.31	− 0.27	− 0.20	0.49
Apr-29	− 0.07	− 0.07	0.33	− 0.21	− 0.20	0.49
Apr-30	− 0.04	− 0.07	0.33	− 0.27	− 0.20	0.49
May-01	− 0.04	− 0.07	0.31	− 0.27	− 0.20	0.49
May-02	− 0.04	− 0.07	0.29	− 0.27	− 0.20	0.50
May-03	− 0.07	− 0.11	0.32	− 0.27	− 0.20	0.50
May-04	− 0.04	− 0.08	0.28	− 0.27	− 0.24	0.54
May-05	− 0.08	− 0.07	0.28	− 0.21	− 0.27	0.57
May-06	− 0.09	− 0.07	0.29	− 0.21	− 0.27	0.57

The left column indicates the beginning of each studied 10-day period

Values in bold are significant correlations

## Discussion

The water quality in the lake during the ten-year study showed great variation, with low pH and high EC and concentrations of Al and sulfate during high runoff events. This reflected well the severe impact from a.s. soils in the area also confirmed in several other studies (Palko and Alasaarela 1988; Palko and Yli-Halla 1993; Roos and Åström 2005; Toivonen and Österholm 2011; Toivonen et al. 2013, 2019). The overall water quality was poorer in the lake than in the Esse River because the three smaller rivers in combination with the numerous low-order streams contributed with a relatively higher acidic metal load (Toivonen and Österholm 2011; Toivonen et al. 2019). Nevertheless, the changes in water quality in the Esse River were found to well represent the changes in the other three rivers in Toivonen and Österholm (2011), and the river is the largest and most important single inflow to the lake (contributes with about 48% of the discharge). Both in the river and the lake, the median pH was roughly equally low during spring as during the autumn, but the most extreme acidity, as well as Al- and sulfate concentrations, was found during the autumn. A severe drop in pH during spring was possibly enhanced by a high input of low-buffered melt water (Laudon and Bishop 1999) commonly taking place during spring, but EC, Al and sulfate concentrations showed less severe levels in spring compared with autumn due to the dilution effect. The lower pH and higher EC found in December and January in the lake compared with the river indicate that the lake reacts slowly on autumn floods, and the acidic water tended to linger in the lake due to the lake retention time. pH was generally high in the summer- and wintertime due to the dominance of a base flow buffered by mineral weathering, cation exchange, etc.) and possibly also photosynthesis performed by aquatic plants and algae during summer.

Aluminum shows high solubility in the acidic conditions found in a.s. soils and is readily flushed to watercourses with harmful ionoregulatory and respiratory effects on the gills of fish due to the high bio availability (Gensemer and Playle 1999; Guéguen et al. 2004; Nystrand and Österholm 2013; Poléo 1995). Compared with the many potentially toxic metals that show the same type of behavior in terms of mobility (e.g., Cd, Ni and Zn), Al is the most abundant in the common minerals (third most common crustal element) and is therefore leached from a.s. soils in the largest quantities (Österholm and Åström 2004; Toivonen et al. 2019). Aluminum is therefore considered to be the key factor in explaining the effects on aquatic biota in acidic environments. Because different species, life stages and populations show different sensitivity to low pH and high Al concentrations, and Al concentrations are reported as different species (e.g., total-, filterable-, inorganic- or labile monomeric form), it is not possible to establish an exact threshold value with harmful pH or Al concentrations. Much work has been produced in obtaining the effects of low pH and high Al concentrations on fish. pH 5.5 and filterable (0.45 µm) Al concentration of 0.5 mg/l is commonly reported as a crude threshold to harmful water quality (Earle and Callaghan 1998). However, Al concentrations in the range of 0.025–0.9 mg/l have been shown to cause disturbance and reduced survival in eggs, juveniles and adult fish in various species even in almost neutral pH (pH 6.8; Hyne and Wilson 1997), but mainly in the interval of 4.0–6.0 (McCahon et al. 1989; Rosseland et al. 1990; Sayer et al. 1993; Vuorinen et al. 1993, Waring and Brown 1995). The conventional filtering (0.45 µm) to obtain the harmful Al concentrations has also proven to be insufficient in the understanding about metal toxicity because colloids carrying metals may pass through the filter and lead to an overestimation of the toxicity of the metals (Nystrand et al. 2012).

In accordance with Nystrand and Österholm (2013), it was shown that a large part of the Al concentrations in the study area was found in bio available small-size fractions as toxic free ions and sulfate complexes. The modeling of the Al species was, however, performed on total Al concentrations, and the large-size and relatively harmless particulate fraction was therefore not accounted for. Nevertheless, the particulate fraction made up about 21% of the total concentrations at pH 5.5, and the fraction is estimated to decrease at decreasing pH. Humic brown-colored water, also typical for the current study area, can be considered to reduce the toxicity of Al to a certain extent due to the ability of organic matter to adsorb toxic elements (Pédrot et al. 2008), making them less bioavailable. According to Vuorinen et al. (1999), waters with high humus content are less toxic despite high Al content than low-humic waters. In line with Nystrand and Österholm (2013), a significant part of Al was complexed to organic matter, but this did not completely remove the toxic fractions.

An extreme acidic surge with following mass kills of fish of many species was observed in the study area, as well as in many other watercourses and coastal areas in Finland, during autumn 2006 and spring 2007. This caused a failure in the reproduction of burbot in the spring of 2007 and 2008. However, even though no visible mass kills of fish has occurred since then, several years with absent or poor occurrence of burbot larvae has been observed in the study area due to the reoccurring poor water quality. The findings in this work indicated that the critical period for the reproduction success for the burbot occurs mainly during the migration of the adult burbot to reproduction sites or during spawning (late December to early February), and possibly also during the early embryonic development stages (February and March). pH proved to be the most crucial for the recruitment of the burbot during January. 10th percentile pH ≤ 5.5, and median pH ≤ 5.8 during January seemed to be able to cause failure in the recruitment of burbot, which was also related to elevated runoff (average runoff > 5 L s^−1^ km^−2^). Even though the common belief is that dry summers are causing intensified acidification following autumn, these results confirm that the acid metal discharge is generally dependent on high runoff conditions, rather than on summer droughts (Toivonen et al. 2013, 2019). Potential difficulties in comparing the larval abundance with the water quality in the lake were caused by spatial patterns in water quality in the lake and its discharging water found in Toivonen and Österholm (2011). The lake is shallow with numerous islands and bays, and the main inflow is in the southern and southeastern part, causing an uneven distribution of the inflowing water. Also, Toivonen and Österholm (2011) found acidic water with high metal concentrations (median pH 4.7 and a median Al concentration of 3.7 mg/l) in the small streams draining into the lake, of which a large part is located in the southeastern part, between Kronoby River and the joint mouth of Esse and Purmo Rivers, close to the sites sampled for burbot larvae. This may also cause inhomogeneous water quality between the two sites sampled for burbot larvae and the site for the industrial water intake. The method therefore requires further tuning, e.g., to what extent or during which tuned time span, the sampling should be performed to obtain sufficient knowledge of the total distribution and effect from acidic metal discharge.

The reoccurring recruitment failure without visible mass kills of fish found in this study is in line with Hudd and Kjellman (2002), who pointed out that it is not only the severity of acidification, but especially the timing of when the lowest pH occurs that has significance for the survival of a year class. Similar effects are also documented for other fish species, and frequently occurring poor recruitment may well have more devastating consequences to fish populations than mass kills of adult fish (Hudd 2000). However, in contrast to this work, Hudd and Kjellman (2002) found the critical period for the survival of a year class to occur around the time of ice breakup and hatching which takes place commonly in April. The discrepancy between the reported critical periods deciding the reproduction success may be due to the warmer winters with rain instead of snow during the 2000s (Toivonen et al. 2013), causing a shift in the timing of the acidic surges from spring toward winter. Since acidity in water courses affected by a.s. soils is highly dependent on runoff, warm winters with prolonged autumn rains may have serious consequences if the expected effects of a climate change comes true (Saarinen et al. 2010).

Even though further tuning of the method is desired (e.g., deciding the threshold value of the critical pH), the overall sensitivity of the burbot to acidic and metal-rich water (Beamish 1976; Bergquist 1991; Rask et al. 1995) and the easy measurement of the abundance of burbot larvae suggests that the method can be useful for evaluating the environmental impact from discharge from a.s. soils. The burbot can be considered as an ideal bioindicator as the timing of migration to spawning sites, spawning and the embryonic and early larval development of the burbot occur mainly during periods when the risk of acidic metal discharge is high (late autumn to spring). The study may also help to cast light on the reasons for the species’ decline and disappearance reported in many European countries (Worthington et al. 2011). Since there are extreme and rapid variations in the temporal acidic metal load (Österholm and Åström 2008; Toivonen et al. 2013, 2019), there is risk of underestimating the load if the assessment is based only on occasional water sampling (Valkama and Ruth 2017; Wallin et al. 2015). In addition, the effect of total concentrations of certain elements, mixtures of metals, concentrations of certain metal species or other water-quality parameters (water hardness, humic substances, etc.) may be difficult to pinpoint when estimating the toxicity of water. Due to the reasons above, a “let the nature speak for itself” approach, advocated in this study, may be highly useful. Using a method highlighting larval abundance also makes it possible to forecast what is going to come in the population and fisheries. The results are commensurable to other measurable variables, e.g., future fish catch statistics, and comprehensible for the fisheries and the broad public. The connection to coastal waters and ecosystems is also clear while using an anadromous fish species to focus on.

## Conclusions

Worldwide, acid sulfate soils have caused lowered chemical and ecological status in many watercourses. The measured pH and metal concentrations during the ten-year study period, as well as the more detailed studies on the complexation behavior and size distribution of Al, confirmed that the water quality frequently dropped to levels that are harmful to the biota during periods of high runoff. The present prediction of climate change indicates warmer winters with rain instead of snow, which may pose an increased hazard in the future to the biota in watercourses affected by a.s. soils. As a species whose biology is sensitive to the timing and severity of acidic metal discharge, the abundance of burbot larvae was in this study shown to have a potential to reflect events with toxic water quality that routine-based water-quality monitoring or analysis may not be able to detect. Due to the episodic nature of the acidic metal discharge, the full ecological consequences from discharge from a.s. soils are not understood with just occasional water sampling. Understanding the full environmental impact from a.s. soils enables application of good indicative monitoring and assessment methods for coastal ecosystems and fish stocks, and encourages the finding of mitigation methods that lead to a more permanent improvement in water quality in the future.
